# A Sensor-Oriented Multimodal Medical Data Acquisition and Modeling Framework for Tumor Grading and Treatment Response Analysis

**DOI:** 10.3390/s26020737

**Published:** 2026-01-22

**Authors:** Linfeng Xie, Shanhe Xiao, Bihong Ming, Zhe Xiang, Zibo Rui, Xinyi Liu, Yan Zhan

**Affiliations:** 1 Department of Biomedical Engineering, Faculty of Engineering, The Hong Kong Polytechnic University, Hong Kong SAR, China; 2 National School of Development, Peking University, Beijing 100871, China; 3 College of Biological Sciences, China Agricultural University, Beijing 100083, China; 4 Artificial Intelligence Research Institute, Tsinghua University, Beijing 100084, China

**Keywords:** medical sensors, multimodal sensing systems, medical imaging sensors, clinical sensing, precision oncology

## Abstract

In precision oncology research, achieving joint modeling of tumor grading and treatment response, together with interpretable mechanism analysis, based on multimodal medical imaging and clinical data remains a challenging and critical problem. From a sensing perspective, these imaging and clinical data can be regarded as heterogeneous sensor-derived signals acquired by medical imaging sensors and clinical monitoring systems, providing continuous and structured observations of tumor characteristics and patient states. Existing approaches typically rely on invasive pathological grading, while grading prediction and treatment response modeling are often conducted independently. Moreover, multimodal fusion procedures generally lack explicit structural constraints, which limits their practical utility in clinical decision-making. To address these issues, a grade-guided multimodal collaborative modeling framework was proposed. Built upon mature deep learning models, including 3D ResNet-18, MLP, and CNN–Transformer, tumor grading was incorporated as a weakly supervised prior into the processes of multimodal feature fusion and treatment response modeling, thereby enabling an integrated solution for non-invasive grading prediction, treatment response subtype discovery, and intrinsic mechanism interpretation. Through a grade-guided feature fusion mechanism, discriminative information that is highly correlated with tumor malignancy and treatment sensitivity is emphasized in the multimodal joint representation, while irrelevant features are suppressed to prevent interference with model learning. Within a unified framework, grading prediction and grade-conditioned treatment response modeling are jointly realized. Experimental results on real-world clinical datasets demonstrate that the proposed method achieved an accuracy of 84.6% and a kappa coefficient of 0.81 in the tumor-grading prediction task, indicating a high level of consistency with pathological grading. In the treatment response prediction task, the proposed model attained an AUC of 0.85, a precision of 0.81, and a recall of 0.79, significantly outperforming single-modality models, conventional early-fusion models, and multimodal CNN–Transformer models without grading constraints. In addition, treatment-sensitive and treatment-resistant subtypes identified under grading conditions exhibited stable and significant stratification differences in clustering consistency and survival analysis, validating the potential value of the proposed approach for clinical risk assessment and individualized treatment decision-making.

## 1. Introduction

Tumor grading and treatment response assessment represent two central challenges in precision oncology, both of which are directly associated with patient prognosis evaluation and treatment planning [1,2,3]. Tumor grading is commonly used to characterize the biological aggressiveness of tumors and serves as an essential basis for assessing disease progression risk and formulating initial therapeutic strategies, whereas treatment response reflects patient sensitivity to interventions such as radiotherapy, chemotherapy, or targeted therapy, and constitutes a key reference for dynamically adjusting treatment regimens to avoid overtreatment or ineffective therapy [4,5,6]. Biologically, tumor grade reflects the degree of cellular differentiation and architectural distortion. High-grade tumors typically exhibit rapid proliferation, significant nuclear atypia, and poor differentiation, which are intrinsically linked to adverse clinical outcomes and resistance to conventional therapies [7,8,9]. Furthermore, grading provides the biological context for treatment response; for instance, high-grade tumors may exhibit higher metabolic activity on PET but simultaneously harbor hypoxic regions that induce radioresistance [10,11]. Therefore, incorporating grading not merely as a label but as a structural prior is essential for decoding the complex heterogeneity of treatment responses [12]. However, real-world clinical practice often shows “different responses within the same grade and similar responses across grades,” where patients with identical tumor grades exhibit heterogeneous treatment responses, while those with different grades may have comparable outcomes under the same regimen [13]. These observations indicate that tumor grading does not directly determine treatment outcomes; rather, its influence is often mediated through a series of intermediate factors, including imaging phenotypes, biological markers, and the overall physiological status of patients [14]. Consequently, traditional treatment decision paradigms that rely solely on tumor grading are increasingly insufficient to meet the dual requirements of individualization and mechanistic interpretability demanded by precision medicine [15].

Advances in medical imaging and clinical information systems enable CT, MRI, and PET to non-invasively characterize tumor spatial architecture, metabolism, and heterogeneity, while clinical phenotypic data complementarily inform patient demographics, pathology, biomarkers, and treatment history [16]. Effective integration of multimodal imaging and clinical phenotypic data, with the aim of achieving non-invasive precise grading while systematically elucidating the mechanisms by which tumor grade influences treatment response, has thus emerged as a critical scientific challenge in precision oncology [17]. At present, pathological biopsy remains the clinical gold standard for tumor grading [18]. Although pathological grading plays an indispensable role in tumor diagnosis, it is highly invasive, limited in sampling scope, and strongly dependent on the experience of pathologists, making it difficult to comprehensively reflect spatial heterogeneity and dynamic tumor evolution [19]. Moreover, repeated biopsies are often infeasible in routine clinical practice, which further constrains the applicability of grading information in treatment response evaluation and longitudinal follow-up [20]. In the context of treatment response assessment, conventional approaches largely rely on imaging-based criteria such as RECIST or on single clinical indicators to determine therapeutic efficacy [21]. Such methods typically focus on macroscopic changes, including tumor size variation, and are therefore limited in their ability to capture early biological alterations within tumors, resulting in suboptimal performance for early response prediction and resistance risk assessment [22]. Meanwhile, tumor grading is incorporated in most response prediction models merely as a static stratification variable rather than being systematically modeled as a key modulating factor influencing treatment response [23]. At the level of multimodal data fusion, traditional studies have predominantly adopted simple feature concatenation or statistical integration strategies, directly feeding imaging features and clinical variables into predictive models [24]. Such coarse-grained fusion approaches neglect the hierarchical relationships and semantic discrepancies among heterogeneous modalities, making it difficult to emphasize features that are highly relevant to tumor grading and treatment sensitivity, and often allowing noisy features to obscure potentially discriminative signals [25].

In recent years, deep learning techniques have achieved remarkable progress in medical image analysis [26,27]. Models based on convolutional neural networks and transformers have been widely applied to tasks such as tumor segmentation, grading prediction, and treatment response assessment, enabling automatic learning of high-dimensional discriminative features from large-scale imaging data [28,29]. Several studies have further integrated radiomics with deep features, leading to improved accuracy in grading or response prediction [30]. Simultaneously, multimodal deep learning frameworks have been increasingly explored to fuse imaging and clinical data in order to enhance overall predictive performance [31]. Vanguri et al. [32] proposed a multimodal prediction model termed DyAM (dynamic attention with masking), which integrates radiological imaging, pathology, and genomic features to more accurately predict the response of patients with non-small cell lung cancer to PD-(L)1 inhibitor immunotherapy. Braman et al. [33] introduced the deep orthogonal fusion (DOF) multimodal framework, which combines MRI, pathology, genomic, and clinical data to enable accurate overall survival prediction in glioma patients, achieving a C-index of approximately 0.788, significantly outperforming single-modality approaches. Tan et al. [34] proposed the MultiCoFusion multimodal framework, which integrates pathological images with gene expression data and performs survival analysis and tumor grade classification simultaneously through multitask correlation learning; improved prognostic prediction and classification performance were demonstrated on TCGA glioma cohorts, validating the effectiveness of multimodal multitask fusion. Wang et al. [35] developed the DeepClinMed-PGM multimodal framework, which integrates pathological images, molecular features, and clinical data for disease-free survival prediction in breast cancer, achieving high predictive performance across multiple cohorts. On the other hand, multimodal fusion strategies generally lack explicit guidance mechanisms, making it difficult for models to distinguish features [36]. In addition, deep models are often limited by insufficient interpretability, and their predictions cannot be readily aligned with established biological mechanisms of tumor progression and therapeutic response, thereby restricting their clinical applicability [37].

Therefore, there is a pressing need for a novel approach that can, within a deep learning framework, jointly integrate multimodal imaging and clinical phenotypic data, treat tumor grading and treatment response as correlated objectives for collaborative modeling, and provide interpretable evidence for medical analysis. This study systematically investigates the core association between tumor grading and treatment response, and the main contributions are summarized as follows:

1. A multimodal deep learning framework for collaborative modeling of tumor grading and treatment response is proposed. This approach unifies grading and response prediction within a single modeling paradigm, aiming to address the limitations of analyzing these correlated factors in isolation.
2. A grade-guided multimodal feature fusion mechanism is designed to selectively highlight imaging and clinical feature representations potentially associated with tumor malignancy and treatment sensitivity, thereby better exploiting the complementary nature of multimodal data.
3. A feature association and interpretation module is constructed to identify and visualize core imaging phenotypes and clinical features associated with treatment-sensitive and treatment-resistant subtypes under different grading contexts. These findings generate data-driven hypotheses regarding the interplay between tumor grade and therapeutic response, providing structured evidence to support further clinical validation.
4. Extensive experiments on real-world clinical datasets demonstrate the effectiveness and robustness of the proposed method in non-invasive grading prediction, treatment response assessment, and survival stratification, highlighting its potential value as a supportive tool for risk assessment in precision oncology.

## 2. Related Work

### 2.1. Research on Solid Tumor-Grading Methods

Tumor grading aims to characterize biological aggressiveness based on cellular differentiation, structural heterogeneity, and invasive behavior [38]. Pathological grading has long been regarded as the gold standard, relying on microscopic assessment of tumor morphology and differentiation to evaluate invasiveness and prognosis [39]. Although clinically essential, this approach depends on invasive biopsy and is highly sensitive to sampling bias and intratumoral heterogeneity, limiting its ability to reflect global tumor characteristics [40]. Moreover, grading outcomes are influenced by pathologist experience, and inter-observer variability remains a non-negligible source of uncertainty [41].

Advances in medical imaging have promoted non-invasive tumor grading based on CT, MRI, and radiomics analysis [42]. By extracting quantitative features describing tumor morphology, texture, and enhancement patterns, imaging-based methods indirectly reflect histological differences [43]. Recent deep learning approaches further improve grading performance through end-to-end representation learning [44], enabling whole-tumor heterogeneity modeling [45]. However, existing studies often focus on single-modality imaging and neglect clinical or molecular information, leading to incomplete biological characterization [46]. In addition, grading is commonly treated as an isolated prediction task, with limited investigation into its impact on treatment response or prognosis, restricting its clinical utility in personalized therapy [47,48].

### 2.2. Tumor Treatment Response Analysis Based on Medical Imaging

Tumor treatment response analysis seeks to predict therapeutic sensitivity and outcomes using imaging and clinical characteristics [49]. Conventional evaluation relies on RECIST criteria, which assess response through tumor size changes [21]. However, such morphology-based indicators often lag behind underlying biological responses, limiting early prediction capability [50]. Consequently, data-driven approaches incorporating radiomic features, imaging phenotypes, and clinical variables have been widely explored [51], based on the assumption that imaging heterogeneity correlates with treatment sensitivity [52]. Deep learning models further enhance feature discovery and have shown promising results across tumor types.

Despite these advances, several challenges persist [53]. Treatment response prediction heavily depends on high-quality labels, which are often compromised by incomplete follow-up and inconsistent evaluation protocols in real-world settings [54]. Moreover, most studies ignore the modulatory role of tumor grading, treating imaging and clinical features independently. As a key indicator of tumor aggressiveness, grading has not been systematically integrated into response modeling, resulting in limited biological interpretability of predictions [55,56].

### 2.3. Multimodal Fusion of Medical Imaging and Clinical Data

Multimodal fusion aims to leverage complementary information from heterogeneous data sources to enhance disease representation [57]. In oncology, imaging captures spatial and biophysical properties, while clinical data reflect systemic physiological states [58]. Existing fusion strategies evolve from input-level to feature-level and decision-level integration [59]. However, most methods adopt general-purpose designs and lack task-specific structural constraints, potentially diluting information critical for grading or treatment response [60,61]. Additionally, simple fusion schemes often fail to model the differentiated contributions of modalities across tasks [62]. Although attention mechanisms improve performance, the biological interpretability and clinical credibility of fused representations remain insufficiently explored [63,64].

### 2.4. Joint Modeling of Tumor Grading and Treatment Response

Studies explicitly modeling the relationship between tumor grading and treatment response remain limited. Most existing work relies on statistical analysis to reveal correlations between grades and outcomes [65,66], without systematically modeling mediating features or causal pathways [67]. Unsupervised subtype discovery approaches often ignore grading as prior knowledge, leading to limited interpretability and weak alignment with clinical grading systems [68]. Overall, an integrated framework linking grading, multimodal features, and treatment response remains lacking; to address this, we propose a grade-guided multimodal collaborative model for interpretable, clinically meaningful precision oncology support.

## 3. Materials and Method

### 3.1. Data Collection

**Figure 1:**
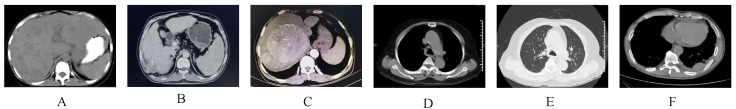
Representative CT imaging examples of different tumor types. (**A**–**C**) Hepatocellular carcinoma (HCC). (**D**–**F**) Non-small cell lung cancer (NSCLC).

The data used in this study were derived from a real-world clinical cohort collected at the oncology center of a tertiary grade A hospital and the Internet, consisting of patients with pathologically confirmed solid tumors, including common tumor types such as non-small cell lung cancer and primary hepatocellular carcinoma, as shown in Table 1 and Figure 1. All enrolled patients received standardized treatment regimens during the study period, including radiotherapy, chemotherapy, or targeted therapy, and were accompanied by complete imaging examinations and structured follow-up records. The patient inclusion period spanned from January 2018 to December 2023, ensuring relative consistency in treatment strategies and imaging equipment conditions. Imaging data were acquired following routine clinical protocols. Specifically, CT data included non-contrast scans and multi-phase contrast-enhanced acquisitions covering arterial, portal venous, and delayed phases, enabling characterization of tumor morphology and vascular supply patterns. MRI data comprised T1-weighted, T2-weighted, and dynamic contrast-enhanced sequences, providing complementary information on soft tissue contrast and intratumoral structural characteristics. PET data were primarily based on ^18^F-FDG PET/CT examinations, with standardized uptake values and other metabolic parameters recorded to reflect tumor metabolic activity and heterogeneity. All imaging data were stored in DICOM format, with complete acquisition parameters and timestamps preserved to support subsequent cross-modality alignment and temporal consistency analysis. Clinical phenotypic data were jointly extracted from the hospital electronic medical record system and tumor-specific clinical databases, including demographic information, pathological indicators, histological subtypes, differentiation grades, and pathological grading results, together with key biomarker data such as PD-L1 expression levels and common driver gene mutation status. Treatment-related information comprehensively documented specific therapeutic regimens, administration cycles, and dosage details. Treatment response evaluation was performed strictly in accordance with RECIST criteria by experienced radiologists, and follow-up outcomes including progression-free survival and overall survival were concurrently collected. All data underwent standardized de-identification procedures after collection, with removal of personal identifiers and anonymization of imaging data. The study protocol was approved by the institutional ethics committee, and written informed consent was obtained from all patients, thereby ensuring compliance with ethical regulations and data security requirements.

From the perspective of data acquisition, the multimodal medical data used in this study can be regarded as being obtained by different types of medical sensor systems. The CT, MRI, and PET imaging processes essentially rely on multichannel physical sensor arrays to capture energy response signals from human tissues. Specifically, CT systems record tissue-dependent X-ray attenuation characteristics through detector arrays, MRI systems employ radiofrequency coils as sensors to acquire nuclear magnetic resonance signals, and ^18^F-FDG PET/CT systems count and localize annihilation photons generated by radiotracers using scintillation crystals and photodetectors. Signal acquisition by these imaging devices is conducted under standardized clinical protocols, and the raw sensor signals are subsequently transformed into spatially consistent medical images through built-in reconstruction algorithms.

Meanwhile, the acquisition of clinical phenotypic and follow-up information primarily depends on hospital information systems and bedside multiparameter monitoring devices, which can be regarded as clinical sensor systems that perform discrete or continuous sampling of patient physiological states and treatment processes. Specifically, vital sign data during hospitalization are collected by bedside multiparameter monitors, including key physiological indicators such as heart rate, blood oxygen saturation, non-invasive blood pressure, respiratory rate, and body temperature. Among these, heart rate, blood oxygen saturation, and respiratory rate are typically recorded in a continuous or quasi-continuous manner, whereas blood pressure and body temperature are obtained through periodic sampling. Signal acquisition is completed using electrocardiographic electrodes, photoplethysmographic sensors, pressure sensors, and temperature sensors, and the raw sensor signals are converted into structured numerical records through embedded algorithms.

In addition, treatment-related process information and follow-up outcomes are collected and aggregated through electronic medical record systems, radiotherapy and chemotherapy management systems, and follow-up databases. These information systems can be regarded as clinical information sensors that perform event-level sampling of diagnostic and therapeutic activities and disease evolution states, covering treatment regimens, medication timing, treatment cycles, response evaluation time points, and survival outcomes. All data generated by the aforementioned clinical sensor systems and information platforms preserve complete timestamps as well as device- or system-level source identifiers, providing the fundamental basis for precise temporal alignment of imaging data, clinical phenotypes, and treatment events, and thereby supporting multimodal joint modeling and temporal consistency analysis.

### 3.2. Data Preprocessing and Augmentation

In multimodal joint modeling tasks involving medical imaging and clinical phenotypes, data preprocessing and augmentation constitute essential prerequisites for ensuring model stability, generalization capability, and cross-modal consistency. Medical imaging data are typically acquired from heterogeneous devices and scanning protocols, while clinical phenotypic data are characterized by strong heterogeneity and high missing rates. Without proper normalization and standardization, systematic biases may be easily introduced, thereby interfering with the effectiveness of collaborative modeling for tumor grading and treatment response. Therefore, prior to model construction, systematic preprocessing was performed separately for medical imaging and clinical data, and targeted augmentation strategies were adopted to enhance model adaptability to complex real-world clinical scenarios. For medical imaging data, spatial resampling was first performed to unify voxel resolutions across patients. Let the original voxel spacing be $\left(s_{x},s_{y},s_{z}\right)$ and the target unified resolution be $\left(s_{x}^{\prime },s_{y}^{\prime },s_{z}^{\prime }\right)$. Through trilinear interpolation, the original image I(x,y,z) was mapped to a new spatial coordinate system $I^{\prime }\left(x^{\prime },y^{\prime },z^{\prime }\right)$, which can be formulated as

(1) $$I^{\prime }\left(x^{\prime },y^{\prime },z^{\prime }\right)=\sum_{i,j,k}I\left(i,j,k\right)\cdot w_{ijk}\left(x^{\prime },y^{\prime },z^{\prime }\right),$$

where $w_{ijk}$ denotes interpolation weights determined by the spatial mapping relationship. This step effectively reduces spatial scale discrepancies caused by different scanning protocols and provides consistent spatial inputs for subsequent deep model learning. After spatial unification, intensity normalization was applied to eliminate gray-level distribution differences induced by variations in imaging devices and acquisition parameters. A distribution-based intensity standardization strategy was adopted, in which each image *I* was transformed to zero mean and unit variance according to

(2) $$I_{norm}=\frac{I-\mu }{\sigma },$$

where μ and σ denote the mean and standard deviation of intensities within tumor-related regions, respectively. This operation enhances robustness to intensity variations and encourages the network to focus on discriminative structural and textural features rather than absolute gray values. To emphasize regions that are closely associated with tumor grading and treatment response, an automatic tumor segmentation model based on deep learning was introduced for region-of-interest extraction. Let the original image be denoted as *I*, and the segmentation model $f_{\theta }\left(\cdot \right)$ output a binary tumor mask *M*. The tumor ROI is then defined as

(3) $$I_{ROI}=I⊙M,$$

where ⊙ represents element-wise multiplication. By focusing on tumor regions, interference from surrounding background tissues is effectively reduced, thereby improving the model’s sensitivity to intratumoral heterogeneity. In addition, to mitigate the influence of imaging noise such as motion artifacts and metal artifacts, artifact suppression was performed using a combination of frequency-domain and spatial-domain techniques to improve overall image quality. For clinical phenotypic data, systematic handling of missing and abnormal values was conducted, as such issues are inevitable in real-world clinical datasets. Missing values were first modeled explicitly. For continuous variables, distribution-based imputation strategies were applied, using feature means or neighborhood-based estimates, whereas mode imputation was adopted for categorical variables. Let *x* denote a continuous clinical variable, and let $x_{miss}$ represent missing values. The imputed value $\hat{x}$ can be expressed as

(4) $$\hat{x}=E\left(x|\Omega \right),$$

where Ω denotes the set of observed samples. This strategy preserves data completeness while minimizing distortion of the underlying variable distribution. After missing value imputation, continuous clinical variables were standardized to eliminate scale discrepancies during model training. The standardization process is defined as

(5) $$x_{std}=\frac{x-\mu_{x}}{\sigma_{x}},$$

where $\mu_{x}$ and $\sigma_{x}$ denote the mean and standard deviation of variable *x* computed from the training set. Categorical variables were transformed using one-hot encoding to facilitate joint learning in deep models. In addition, outlier detection based on statistical distributions and boxplot principles was employed to correct or remove clinical records that deviated substantially from normal ranges, thereby improving overall data quality. To further enhance generalization performance under limited sample conditions, multi-strategy augmentation mechanisms were introduced at both imaging and clinical phenotype levels. At the imaging level, random spatial transformations were applied to tumor ROIs to simulate variability in clinical imaging conditions, including random flipping, rotation, noise perturbation, and elastic deformation. Let the augmentation operation be represented by a random transformation operator $T_{\alpha }$, where α denotes transformation parameters. The augmented image is then given by

(6) $$I_{aug}=T_{\alpha }\left(I_{ROI}\right).$$

This strategy expands the feature distribution space while preserving tumor semantic consistency, enabling the model to learn more robust features related to grading and treatment response. At the clinical phenotype level, substantial imbalance often exists among different combinations of tumor grade and treatment response. To alleviate insufficient learning of minority subtypes, a synthetic minority over-sampling strategy was adopted. The core idea is to generate new synthetic samples through linear interpolation in the feature space. Let $x_{i}$ denote a minority class sample and $x_{j}$ its nearest neighbor. A synthetic sample $x_{new}$ is generated as

(7) $$x_{new}=x_{i}+\lambda \left(x_{j}-x_{i}\right),$$

where λ∈(0,1) is a random interpolation coefficient. This approach increases the representation of minority grade–response subtypes during training without introducing excessive noise. Furthermore, considering the importance of temporal consistency across modalities for collaborative modeling, a cross-modal temporal alignment strategy was incorporated during preprocessing. Specifically, imaging data and corresponding clinical phenotypic records were required to be strictly matched within a defined temporal window, such that the imaging acquisition time $t_{img}$ and the clinical record time $t_{cli}$ satisfy

(8) $$|t_{img}-t_{cli}|\;\leq \;\delta ,$$

where δ denotes a predefined temporal tolerance threshold. Temporal alignment effectively prevents potential biases caused by disease progression or treatment stage mismatch, ensuring that multimodal features are jointly modeled under the same biological state.

### 3.3. Proposed Method

#### 3.3.1. Overall

After data alignment and normalization, the proposed method adopts dual-modality inputs of medical imaging and clinical phenotypes as the starting point, and constructs an end-to-end closed-loop pipeline encompassing feature extraction, grade-guided fusion, collaborative learning, and mechanism interpretation at the model design level. Specifically, given the multimodal imaging tensor set $\left\{X^{ct},X^{mri},X^{pet}\right\}$ and the clinical phenotype vector $X^{cli}$ for each patient, the data are first fed into the associated feature extraction module.

On the imaging side, a hybrid CNN–Transformer architecture is specifically adopted as the backbone to map different imaging modalities into a unified latent space, yielding the imaging representation $Z^{img}=E_{img}\left(\left\{X^{ct},X^{mri},X^{pet}\right\}\right)$. This architectural choice is theoretically grounded in the inherent hierarchical structure of medical imaging data: convolutional layers are utilized to extract local texture features and anatomical patterns, while the Transformer’s self-attention mechanism is subsequently applied to capture long-range global dependencies and cross-regional interactions. Within this shared representation, a grading-oriented sub-representation $Z^{g}$ and a treatment-sensitivity-oriented sub-representation $Z^{r}$ are further disentangled, while an interaction mapping is applied to obtain the cross-associated representation $Z^{gr}$, which captures coupling patterns between malignancy degree and treatment sensitivity.

On the clinical side, structured encoding and feature selection networks are used to map $X^{cli}$ into $Z^{cli}=E_{cli}\left(X^{cli}\right)$, and correlation constraints are imposed to align it with the imaging latent space, ensuring that grading- and response-related clinical variables can be seamlessly integrated into subsequent modules. Subsequently, the grade-guided fusion module takes the pathological grade $y^{g}$ as a weakly supervised structural prior and combines it with the treatment response status $y^{r}$ to construct a joint guidance signal $u=\Phi (y^{g},y^{r})$. Guided attention is then applied to dynamically weight $\left\{Z^{g},Z^{r},Z^{gr},Z^{cli}\right\}$, forming the fused representation $Z^{fus}=F\left(\left\{Z^{g},Z^{r},Z^{gr},Z^{cli}\right\},u\right)$. This guidance mechanism enables the model to automatically emphasize feature channels most relevant to response heterogeneity under different grading contexts while suppressing irrelevant noise. Based on $Z^{fus}$, a collaborative modeling network is constructed with a shared backbone and dual-branch architecture. The grading branch outputs ${\hat{y}}^{g}=H_{g}\left(Z^{fus}\right)$ and aligns it with $y^{g}$ to achieve non-invasive grading prediction, whereas the response branch performs representation aggregation and clustering on $Z^{fus}$ under grading conditions to learn latent subtype assignments $\pi =H_{r}\left(Z^{fus}\right)$, facilitating the discovery of sensitive and resistant subgroups and enhancing separability by associating subtypes with follow-up response signals. Finally, the mechanism interpretation module maps the learned attention weights and feature contributions back to the original feature domain, forming a set of key mediating features *S*, and analyzes the association pathways between *S* and subtypes or responses under grading constraints. This process yields an interpretable transmission chain of “grading–core features–treatment response,” providing structured evidence for subsequent clinical validation and decision support.

#### 3.3.2. Grading–Treatment Response Associated Feature Extraction Module

**Figure 2:**
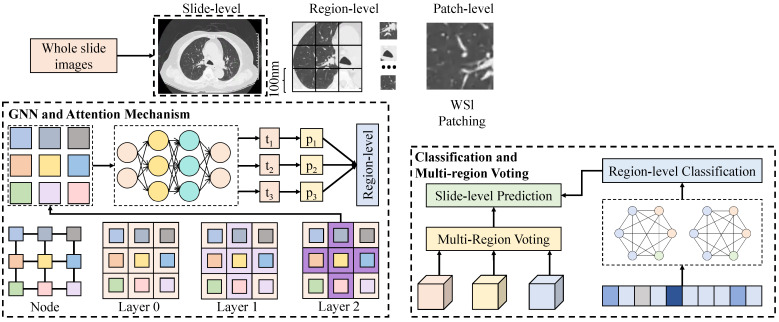
Illustration of the Grading–Treatment Response Associated Feature Extraction Module.

The grading–treatment response associated feature extraction module takes multiscale pathological imaging and structured clinical phenotypes as joint inputs, with the primary objective of explicitly characterizing shared and distinct representations between tumor malignancy grading and treatment sensitivity at the feature level. This design provides biologically oriented high-quality features for subsequent grade-guided fusion and collaborative modeling. In implementation, the imaging branch adopts a hierarchical multiscale modeling strategy consistent with the module architecture, progressively abstracting tumor phenotypes from whole-slide images to local tissue units. As shown in Figure 2, whole-slide images are used as input, and a set of high-resolution candidate regions is obtained via random region sampling at the slide-level stage to avoid over-reliance on local salient regions while neglecting global heterogeneity. At the region-level stage, each region is further divided into subregions with fixed physical scales, serving as basic units for modeling mesoscopic tissue structures. Subsequently, at the patch-level stage, regions are decomposed into small patches and fed into a shared-weight convolutional encoder, whose backbone consists of multiple convolutional and normalization layers to output high-dimensional representations for each patch. To enhance the modeling of long-range spatial dependencies, a lightweight Transformer encoding module is introduced on top of the patch features, where self-attention mechanisms capture relationships among different tissue regions. The resulting patch-level features are further aggregated into unit-level representations corresponding to key histological components such as tumor cells, stroma, vasculature, immune cells, and necrotic regions, which can be regarded as embeddings of different pathological constituents in the latent space.

Within this hierarchical structure, let the initial feature of the *i*-th patch be denoted as $z_{i}^{p}$. After self-attention encoding, the feature becomes $z_{i}^{a}$, and the region-level representation is obtained via weighted aggregation as $z^{r}=\sum_{i}\alpha_{i}z_{i}^{a}$, where the weights $\alpha_{i}$ are adaptively learned by the attention network to highlight local tissue patterns relevant to grading or treatment response. Based on this representation, a feature disentanglement mapping is introduced to project $z^{r}$ into two subspaces, yielding the grading-related feature $z^{g}=W_{g}z^{r}$ and the treatment-response-related feature $z^{t}=W_{t}z^{r}$, where $W_{g}$ and $W_{t}$ are learnable linear transformation matrices. To capture interactions between these two aspects, a cross-associated feature $z^{gt}=z^{g}⊙z^{t}$ is further constructed to represent the coupling patterns between malignancy degree and treatment sensitivity at the imaging level. From a mathematical perspective, this design explicitly introduces second-order interaction terms into the feature space, enabling the model to learn complex patterns such as “high grade–high heterogeneity–high sensitivity” or “low grade–low metabolism–resistance,” rather than relying solely on linear discrimination. On the clinical feature side, a combination of multilayer perceptrons and graph-based modeling is employed for encoding. Raw clinical variables are first mapped into continuous vectors through an embedding layer and then processed by a stack of fully connected layers to obtain the initial clinical representation $z^{c}$. Considering the potential dependencies among clinical variables, a graph convolution-based relational modeling module is introduced, where each variable is treated as a node and an adjacency matrix encodes statistical correlations among variables, resulting in a structured clinical representation $z^{c^{\prime }}$. This representation is subsequently aligned with the imaging-side features $z^{g}$ and $z^{t}$ through correlation constraints, ensuring semantic consistency between clinical and imaging features in the latent space.

After joint encoding of imaging and clinical features, the module performs feature selection at the final stage using mutual information maximization and redundancy suppression strategies, retaining feature dimensions that contribute substantially to both grading prediction loss and treatment response association. Overall, this module ensures that grading-related and treatment-sensitivity-related features are both distinguishable and interrelated through multiscale imaging modeling, feature disentanglement, and interaction design. From a structural perspective, it guarantees explicit separation and coupling of task-relevant representations; from a mathematical perspective, it enhances discriminative capacity via subspace projection and multiplicative interactions; and from a task perspective, it effectively mitigates the long-standing issue of isolated modeling of grading and treatment response, providing biologically meaningful intermediate representations for subsequent grade-guided fusion and mechanism interpretation.

#### 3.3.3. Grade-Guided Multimodal Fusion Mechanism

**Figure 3:**
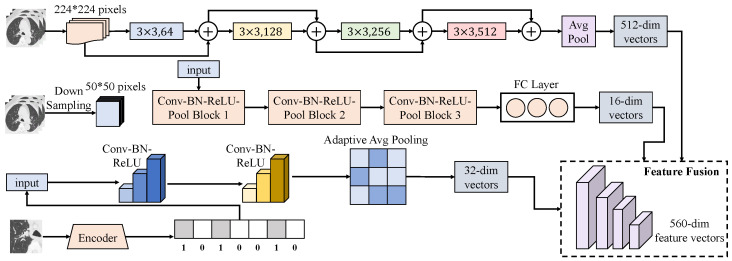
Illustration of the grade-guided multimodal fusion mechanism.

The grade-guided multimodal fusion mechanism is activated after completing multi-branch encoding on the imaging side and clinical phenotype encoding. Its input consists of three imaging representations and one clinical representation, while grading information is incorporated as a weakly supervised structural prior to dynamically regulate fusion weights. Specifically, the imaging branch is organized into three parallel feature streams according to the architectural design. As shown in Figure 3, the ResNet branch receives 224×224 patch inputs and produces a 512-dimensional vector $h^{res}\in R^{512}$ through a ResNet34 backbone followed by global average pooling. The Direct branch downsamples patches to 50×50 and processes them through three Conv-BN-ReLU-Pool blocks, each using 3×3 convolutional kernels with channel dimensions increasing from 8 to 16 to 32, and 2×2 pooling with stride 2, after which two fully connected layers map the spatial features to a 16-dimensional vector $h^{dir}\in R^{16}$. The Encoded branch takes unit-level inputs of size 20×20×7, applies two successive 3×3 convolutions to obtain feature maps of size 20×20×16 and 18×18×32, and then compresses them via adaptive average pooling to 1×1×32, yielding a flattened 32-dimensional vector $h^{enc}\in R^{32}$. The clinical phenotype vector $h^{cli}$ is generated by an independent encoder and projected to 64 dimensions to match the fusion scale. These representations are concatenated to form the base feature vector $h=\left[h^{res};h^{dir};h^{enc};h^{cli}\right]\in R^{512+16+32+64}=R^{624}$.

To inject grading priors into the fusion process, the pathological grade label $y^{g}$ is first mapped through a learnable embedding layer to produce a guidance vector $g\in R^{d_{g}}$, with $d_{g}=32$ in the implementation, which is further combined with the response status embedding to form a conditional context. Rather than static concatenation, a conditional gated attention mechanism is employed in the fusion layer. Each modality-specific feature is linearly aligned to a unified dimension D=128 as ${\tilde{h}}^{m}=W_{m}h^{m}+b_{m}$, where m∈{res,dir,enc,cli}. The guidance vector then modulates the weighting process, yielding

(9) $$s_{m}=v^{⊤}\tanh\left(A{\tilde{h}}^{m}+Bg+c\right),\;\alpha_{m}=\frac{\exp(s_{m})}{\sum_{k}\exp\left(s_{k}\right)},$$

(10) $$z=\sum_{m}\alpha_{m}{\tilde{h}}^{m},\;z^{fus}=\phi \left(W_{f}z+b_{f}\right),$$

where ϕ(·) denotes the ReLU activation and $z^{fus}\in R^{D}$ serves as the fused representation fed into the shared backbone of the collaborative modeling network. To preserve the high-capacity complementary information emphasized in the multi-branch design, a residual pathway retains the original imaging vectors and concatenates them with $z^{fus}$ to produce the final fused vector $u^{fus}\in R^{560}$, corresponding to the combined imaging dimensions 512+16+32, with clinical dimensions optionally appended in parallel during implementation. When jointly used with the grading–treatment response collaborative modeling network, the grading branch predicts ${\hat{y}}^{g}$ from $u^{fus}$, while the response subtype branch learns conditional clustering structures from $z^{fus}$, thereby sharing a consistent grading context under the same conditional attention. The advantage of grade-guided fusion can be illustrated by noting that *z* is a convex combination of modality-aligned representations satisfying $\sum_{m}\alpha_{m}=1$ and $\alpha_{m}\geq 0$. If each modality is decomposed into signal and noise components as ${\tilde{h}}^{m}=r^{m}+\epsilon^{m}$ with independent zero-mean noise $\epsilon^{m}$, the variance of the fused noise is bounded by

(11) $$\mathrm{Var}\left(\sum_{m}\alpha_{m}\epsilon^{m}\right)=\sum_{m}\alpha_{m}^{2}\;\mathrm{Var}\left(\epsilon^{m}\right)\leq \sum_{m}\alpha_{m}\;\mathrm{Var}\left(\epsilon^{m}\right),$$

where the inequality follows from $0\leq \alpha_{m}\leq 1$, indicating that guiding the weights toward more reliable modalities further suppresses noise energy. To enhance controllability of the guidance signal over weight allocation, an entropy regularization term is introduced to maintain dispersed attention for uncertain samples and more focused attention for samples with clear grading evidence:

(12) $$L_{gate}=\gamma \;E\left[-\sum_{m}\alpha_{m}\log\alpha_{m}\right].$$

When jointly optimized with grading supervision loss and response stratification objectives, this formulation theoretically improves both non-invasive grading stability and separability of sensitive and resistant subtypes under grading constraints by simultaneously reducing noise bounds and enhancing conditional discriminability. Consequently, fusion becomes a task-customized representation with explicit grading semantics rather than a generic concatenation, providing clearer and traceable feature contribution pathways for subsequent mechanism interpretation.

#### 3.3.4. Grading–Treatment Response Collaborative Modeling and Mechanism Interpretation

**Figure 4:**
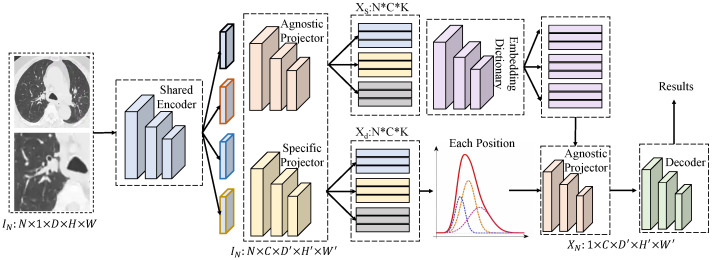
Illustration of the grading–treatment response collaborative modeling and mechanism interpretation framework.

The grading–treatment response collaborative modeling and mechanism interpretation module is activated after obtaining the grade-guided fused representation. Its design follows the unified pipeline illustrated in the architecture, comprising shared encoding, generic and specific projections, dictionary querying and reconstruction, adaptive assembly, and decoding or discrimination, thereby completing a closed loop for grading prediction, response subtype learning, and interpretable mechanism tracing within a single representation space. As shown in Figure 4, the fused imaging–clinical input is organized into two forms. The first is a spatial feature tensor $F\in R^{N\times C\times D^{\prime }\times H^{\prime }\times W^{\prime }}$ produced by the multimodal imaging branches, where C=128, $D^{\prime }=8$, $H^{\prime }=32$, and $W^{\prime }=32$, generated by a shared encoder. The second is a global vector $c\in R^{N\times 64}$ obtained by projecting clinical phenotypes, which provides patient-level conditional context.

The shared encoder adopts a 3D convolutional backbone with four stages, each consisting of two 3×3×3 convolutions and residual connections. Channel dimensions increase progressively from 32 to 64, 96, and 128, while spatial resolution is downsampled by stride 2 to $\left(D^{\prime },H^{\prime },W^{\prime }\right)$, enabling the capture of both local texture sensitivity and global structural information. The encoded tensor *F* is then processed by two parallel projection heads. The generic projector $P_{a}$ comprises two 1×1×1 convolutions that map channels from 128 to 128 and then to $C_{e}=64$, yielding a position-invariant embedding sequence $X_{s}\in R^{N_{p}\times C_{e}}$. The specific projector $P_{d}$ shares a similar structure but incorporates conditional affine modulation generated from *c*, producing $X_{d}\in R^{N_{p}\times C_{e}}$ to emphasize response-relevant components under grading contexts, where $N_{p}=D^{\prime }H^{\prime }W^{\prime }=8192$ denotes the number of spatial positions. A learnable embedding dictionary $E\in R^{K\times C_{e}}$ with K=256 is introduced for querying and reconstruction. For each position, the query vector $q_{i}=X_{s}\left(i\right)$ is matched with the dictionary to obtain sparse coefficients and reconstruct the generic representation ${\tilde{x}}_{i}$ as

(13) $$a_{i}=\mathrm{softmax}\left(\frac{q_{i}E^{⊤}}{\tau }\right),\;{\tilde{x}}_{i}=a_{i}E,$$

where τ denotes a temperature parameter. To decouple generic structural information from response-specific cues, the specific branch does not directly reconstruct features. Instead, it learns a position-wise sampling gate $g_{i}\in \left(0,1\right)$ generated from $X_{d}\left(i\right)$ via a two-layer MLP with hidden dimension 32, and adaptively assembles the reconstruction residual to obtain the collaborative representation $y_{i}$:

(14) $$g_{i}=\sigma \left(W_{2}\phi \left(W_{1}X_{d}\left(i\right)\right)\right),\;y_{i}={\tilde{x}}_{i}+g_{i}⊙\left(X_{d}\left(i\right)-{\tilde{x}}_{i}\right).$$

The set $\left\{y_{i}\right\}$ is reshaped back to $Y\in R^{N\times C_{e}\times D^{\prime }\times H^{\prime }\times W^{\prime }}$, after which a lightweight 3D upsampling decoder with three stages and channel progression 64→48→32→16 reconstructs key tumor structures at resolution D×H×W. This reconstruction provides visual support for mechanism interpretation and constrains the representation to preserve biologically meaningful morphology. Collaborative modeling adopts a dual-head strategy on the shared representation. The grading head applies global average pooling to *Y* to obtain $u\in R^{N\times 64}$, followed by two fully connected layers (64→32→G, where *G* denotes the number of grading classes) to output ${\hat{y}}^{g}$. The response subtype head performs prototype-based clustering on *u*, learning *M* subtype centers ${\left\{\mu_{m}\right\}}_{m=1}^{M}$ with M=2 or 3, and produces soft assignments π as

(15) $$\pi_{m}=\frac{\exp(-∥u-\mu_{m}{∥}_{2}^{2}/\kappa )}{\sum_{j=1}^{M}\exp(-∥u-\mu_{j}{∥}_{2}^{2}/\kappa )}.$$

The overall objective jointly optimizes grading supervision, subtype compactness, and structural reconstruction:

(16) $$L=L_{grade}+\lambda \sum_{m}\pi_{m}∥u-\mu_{m}{∥}_{2}^{2}+\beta {∥F-\hat{F}∥}_{1},$$

where $\hat{F}$ denotes the reconstructed features. The key advantage of this design lies in the controllable generic basis provided by dictionary reconstruction, which encourages $X_{s}$ to capture grading-stable morphological commonalities, while the gated assembly introduces specific deviations only when necessary. Consequently, the collaborative representation can be expressed as $y_{i}={\tilde{x}}_{i}+g_{i}⊙\Delta_{i}$ with $\Delta_{i}=X_{d}\left(i\right)-{\tilde{x}}_{i}$. When $g_{i}$ approaches 0 at noisy locations, $y_{i}$ reduces to the stable generic reconstruction, preventing the response branch from being driven by spurious variations; when $g_{i}$ approaches 1 at key pathological structures, specific cues are retained to enhance subtype separability. Mechanism interpretation is achieved by propagating $g_{i}$ and $\pi_{m}$ back to the spatial domain to construct subtype-sensitive maps $S_{m}\left(p\right)=E\left[g_{p}\pi_{m}\right]$, which are further correlated with clinical biomarkers to form a traceable chain of “grading–mediating structures–subtypes–response.” In the context of the present task, this collaborative modeling strategy simultaneously improves non-invasive grading consistency, enhances separability of response subtypes under grading constraints, and provides spatially grounded evidence for subsequent biological validation.

## 4. Results and Discussion

### 4.1. Experimental Configuration

#### 4.1.1. Hardware and Software Platform

All experiments were conducted on a high-performance GPU server. The computing nodes were equipped with multi-core CPUs and large-capacity memory to meet the high computational and storage demands of multimodal medical imaging data during preprocessing, feature extraction, and model training. GPUs with deep learning acceleration capabilities and sufficient memory capacity were employed to accommodate multimodal three-dimensional images and batch-based training simultaneously, thereby ensuring the stable execution of complex models such as 3D convolutional networks and transformers. The overall hardware environment effectively supported repeated experiments, cross-validation, and ablation studies, avoiding training instability or result bias caused by computational bottlenecks. On the software side, the experimental system was built on a Linux operating system. Model implementation and training were carried out using the PyTorch 1.9.0 deep learning framework, with CUDA and cuDNN utilized for GPU-accelerated computation. Medical image processing operations were performed using open-source libraries such as SimpleITK and Nibabel, including image loading, resampling, normalization, and ROI cropping. Statistical analysis and preprocessing of clinical phenotypic data were mainly implemented using NumPy, Pandas, and Scikit-learn within the Python 3.8 ecosystem, while survival analysis and visualization were conducted with Lifelines and Matplotlib 3.9.8. This software environment ensured the reproducibility and standardization of the experimental pipeline.

#### 4.1.2. Hyperparameter Settings

For dataset partitioning, the complete dataset was randomly split at the patient level to prevent information leakage. Specifically, 70% of the data were used for model training, 15% were reserved for hyperparameter tuning and model selection, and the remaining 15% constituted an independent test set for final performance evaluation. In addition, to further assess the robustness of the results, a five-fold cross-validation strategy was employed during training. The training data were evenly divided into five non-overlapping subsets, with one subset used as the validation set and the remaining subsets used for training in each fold. The average performance across the five folds was reported as the final evaluation result. During model training, the imaging branches and the clinical branch were optimized jointly in an end-to-end manner. Regarding the multi-task loss function configuration, the weighting coefficients λ and β were determined through a coarse-to-fine grid search on the validation set. Specifically, to balance the gradient magnitudes between the supervised grading loss and the unsupervised regularization terms, λ was empirically set to 0.1 to control subtype compactness, while β was set to 0.05 to constrain reconstruction fidelity. This allocation ensures that the auxiliary unsupervised tasks provide structural constraints without dominating the primary grading objective. The initial learning rate was set to $\alpha =1\times 10^{-4}$, and an adaptive decay strategy based on validation performance was applied. To address potential discrepancies in convergence speeds between the tumor grading and treatment response tasks, a task-aware monitoring strategy was implemented. While the learning rate decay was primarily triggered by the plateauing of the grading accuracy, the early stopping mechanism required the stabilization of both grading metrics and the response subtype clustering consistency. In cases where the grading branch converged significantly earlier than the response branch, the parameters of the grading head were frozen, and the shared encoder along with the response branch were fine-tuned for additional epochs with a reduced learning rate (0.1×α) to ensure sufficient convergence of the latent subtype discovery process. The batch size was determined according to GPU memory capacity to balance training stability and efficiency. The Adam optimizer was adopted with momentum parameters $\beta_{1}=0.9$ and $\beta_{2}=0.999$, and a weight decay coefficient was applied to mitigate overfitting. These hyperparameter configurations demonstrated stable performance across multiple experiments and provided a reliable training foundation for collaborative modeling of tumor grading and treatment response.

### 4.2. Baseline Models

To systematically evaluate the performance of the proposed approach in collaborative modeling of tumor grading and treatment response, a series of classical models that have been widely adopted in medical image analysis and clinical modeling were selected as comparative baselines, covering single-modality, multimodal, and deep fusion paradigms. For the tumor-grading task, a 3D resnet-18 model based on ct imaging [69] was first adopted as the single-modality imaging baseline. This model is capable of effectively modeling the spatial structure and morphological characteristics of tumors through three-dimensional convolutions and has demonstrated stable and representative performance in medical image grading tasks. In parallel, a multilayer perceptron (MLP) based on clinical phenotypes [70] was employed as the clinical single-modality grading model to characterize the discriminative capacity of structured variables, including demographic information, biological markers, and pathological indicators. For multimodal grading, a traditional early-fusion baseline, namely 3D resnet + MLP [71], was introduced, in which imaging and clinical features are jointly modeled through feature-level concatenation. In addition, to assess the capability of deep multimodal representation learning, a cnn–transformer multimodal model without grading guidance [29] was included to capture high-order associations across heterogeneous modalities. For treatment response prediction and subtype analysis, a 3D resnet-18 model based on pet imaging [69] was adopted as the single-modality metabolic response prediction baseline, while a gaussian mixture model (GMM) driven by clinical biomarkers [72] was utilized for unsupervised response subtype discovery. In the multimodal response modeling setting, the same 3D resnet + MLP early-fusion model [71] and the cnn–transformer multimodal model [29] were employed as comparative methods. Through these baseline configurations, the effectiveness and advantages of the proposed grade-guided collaborative modeling framework were validated from multiple perspectives.

### 4.3. Evaluation Metrics

For grading accuracy assessment, classification accuracy was used to measure the proportion of agreement between predicted grades and pathological grades, while the kappa coefficient was employed to evaluate the reliability of the agreement beyond chance. For treatment response prediction, the area under the receiver operating characteristic curve (AUC) was used to reflect overall discriminative ability across different thresholds, and precision and recall were jointly reported to assess the accuracy and completeness of identifying treatment-sensitive and resistant patients. In subtype analysis, clustering consistency metrics were used to quantify the stability of subtype assignments across repeated experiments or subsampled datasets. In survival analysis, the concordance index (C-index) was adopted to measure the agreement between predicted risk rankings and actual survival times, and Kaplan–Meier survival curves were used to compare survival differences among different grade–subtype combinations. In addition, a clinical interpretability score was introduced to quantitatively assess the consistency between model-derived features and established clinical or biological knowledge. The mathematical definitions of the evaluation metrics are given as follows:

(17) $$\mathrm{Accuracy}=\frac{TP+TN}{TP+TN+FP+FN},$$

(18) $$\kappa =\frac{p_{o}-p_{e}}{1-p_{e}},$$

(19) $$\mathrm{Precision}=\frac{TP}{TP+FP},\;\mathrm{Recall}=\frac{TP}{TP+FN},$$

(20) $$\mathrm{AUC}=\int_{0}^{1}\mathrm{TPR}\left(\alpha \right)\;d\mathrm{FPR}\left(\alpha \right),$$

(21) $$C\text{-}\mathrm{index}=\frac{1}{\left|P\right|}\sum_{(i,j)\in P}I\left({\hat{r}}_{i}>{\hat{r}}_{j}\right),$$

where TP, TN, FP, and FN denote the numbers of true positives, true negatives, false positives, and false negatives, respectively. The term $p_{o}$ represents the observed agreement rate, and $p_{e}$ denotes the expected agreement by chance. The functions TPR(α) and FPR(α) correspond to the true positive rate and false positive rate at threshold α, respectively. The set P contains all comparable sample pairs, ${\hat{r}}_{i}$ and ${\hat{r}}_{j}$ denote the predicted risk scores for individuals *i* and *j*, and I(·) is the indicator function.

### 4.4. Results on Grading and Treatment Response Modeling

This section provides a systematic evaluation of the effectiveness and advantages of the proposed framework in tumor grading and treatment response collaborative modeling tasks. Rather than solely pursuing the optimal value of a single performance metric, the experimental design is driven by two clinically critical stages, namely diagnosis and treatment, with the aim of verifying the structural role of grading information in multimodal modeling. Specifically, the experiments examine whether tumor grading can serve as an intermediate variable that effectively organizes heterogeneous information from imaging and clinical features, thereby simultaneously improving the consistency of non-invasive grading prediction and the stability of treatment response modeling.

**Figure 5:**
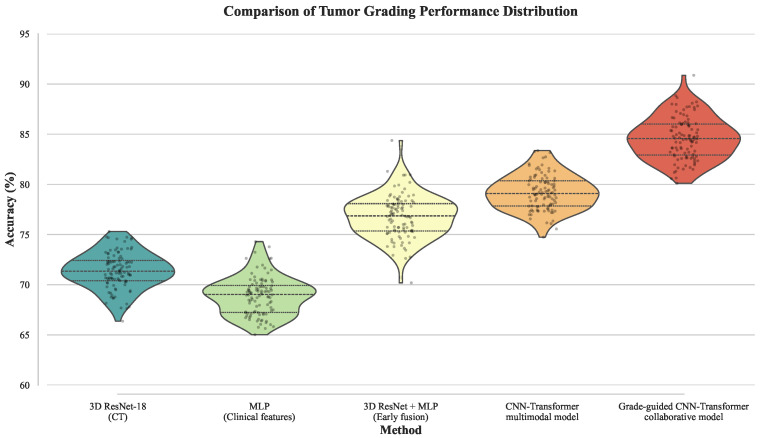
Comparison of tumor-grading performance.

Based on these objectives, the experiments first focus on tumor-grading performance, and a systematic comparison is conducted across different modeling paradigms for malignancy discrimination, as summarized in Table 2 and Figure 5. It can be observed that single-modality grading models exhibit limited performance in terms of both accuracy and consistency. The CT-based 3D ResNet-18 is capable of effectively capturing three-dimensional morphological structures of tumors; however, the absence of holistic clinical context constrains its discriminative capacity. In contrast, the MLP-based clinical model leverages demographic variables and biomarkers, but its coarse-grained feature representation limits the characterization of intratumoral spatial heterogeneity. When imaging and clinical features are combined through early fusion in the 3D ResNet + MLP model, grading performance is noticeably improved, demonstrating the complementary value of multimodal information. Nevertheless, this strategy relies on simple feature concatenation and lacks explicit differentiation across modalities and semantic levels. The CNN–Transformer multimodal model further improves grading performance, indicating that deep cross-modal interaction facilitates the learning of more discriminative joint representations, yet explicit grading constraints are still absent. Ultimately, the proposed grade-guided CNN–Transformer collaborative model achieves the best performance in both accuracy and Kappa coefficient. This result suggests that injecting grading information as a weakly supervised structural prior into the multimodal fusion process enables the formation of clearer and more stable class boundaries in high-dimensional feature space, thereby enhancing the consistency of non-invasive grading prediction.

**Figure 6:**
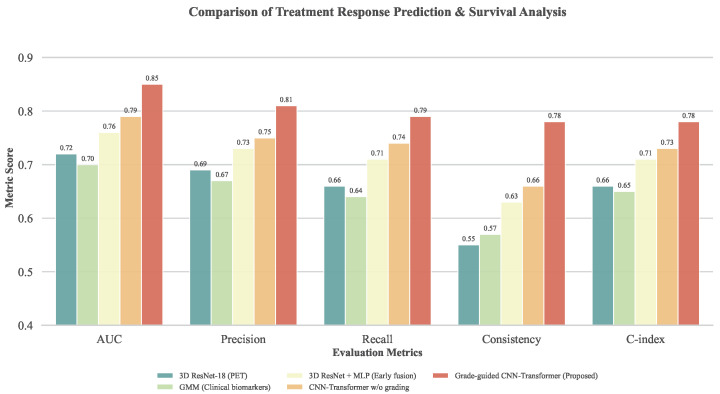
Comparison of treatment response prediction, subtype stability, and survival analysis.

After validating grading performance, the analysis is further extended to treatment response prediction, subtype stability, and survival analysis, which are more closely aligned with clinical decision-making. The corresponding results are reported in Table 3 and Figure 6. The objective of this experiment is to examine whether grading information contributes not only at the diagnostic stage but also acts as a critical conditional variable in treatment modeling. Overall, single-modality response models show limited performance across AUC, precision, and recall. The PET-based 3D ResNet-18 primarily relies on metabolic activity signals, while the GMM based on clinical biomarkers captures only partial molecular-level variations; neither approach sufficiently represents tumor spatial heterogeneity and inter-patient variability. The early fusion 3D ResNet + MLP model achieves more stable improvements across metrics, confirming the benefit of multimodal integration, although subtype consistency and survival relevance remain constrained. The CNN–Transformer model without grading guidance further enhances response prediction and survival analysis, demonstrating the effectiveness of deep cross-modal modeling. However, the proposed grade-guided CNN–Transformer collaborative model consistently outperforms all baselines across all metrics, with particularly notable advantages in subtype consistency and C-index. These findings indicate that incorporating grading as a conditional constraint effectively reduces interference across samples from different grades, enabling the model to learn more stable and biologically meaningful response structures within grade-specific contexts, thereby improving both treatment response prediction and prognostic stratification.

### 4.5. Mechanism Analysis and Clinical Interpretation

In this section, the primary objective is not merely to compare predictive performance, but to systematically verify whether the proposed grade-guided collaborative framework, built upon established modeling paradigms such as 3D ResNet-18, MLP, and CNN–Transformer multimodal models, can further identify key phenotypes that are highly consistent with tumor grading and exhibit clear associations with treatment response. In contrast to the previous section, which emphasizes quantitative performance, this section focuses on whether internal model representations form stable and interpretable associations linking grading, features, and treatment response.

**Figure 7:**
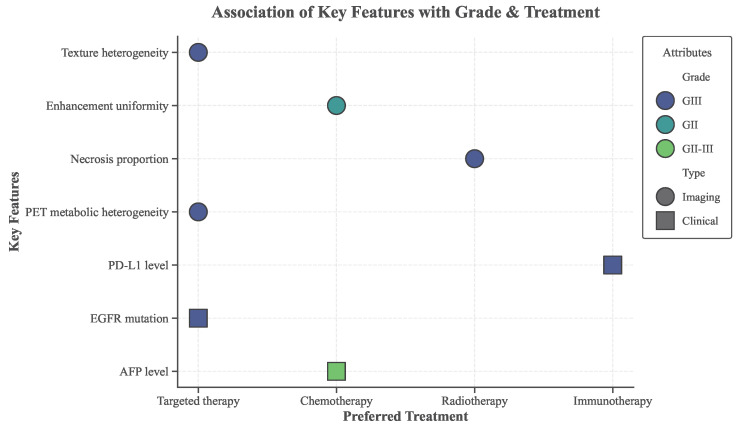
Key imaging and clinical features associated with tumor grading and treatment preference.

Based on the attention distributions and feature contribution patterns learned by the grade-guided CNN–Transformer collaborative model, high-weight features are statistically analyzed from both imaging phenotypes and clinical biomarkers, with results summarized in Table 4 and Figure 7. Distinct stratification patterns across grades are observed, and these patterns exhibit strong consistency with commonly adopted clinical treatment strategies. In high-grade tumors (GIII), texture heterogeneity, necrotic proportion, and PET metabolic heterogeneity captured by 3D ResNet-18 and CNN–Transformer jointly reflect pronounced spatial and metabolic nonuniformity, and these features are preferentially associated with targeted therapy or radiotherapy. In contrast, tumors at intermediate grade (GII) exhibit more uniform enhancement patterns, which align with chemotherapy strategies that depend on vascular perfusion and drug distribution. On the clinical side, PD-L1 expression and EGFR mutation status encoded by the MLP and aligned within the multimodal space are predominantly enriched in GIII cases, and their corresponding immunotherapy and targeted therapy options are consistent with established oncological knowledge. These observations indicate that, under grading constraints, the learned features reconstruct a stable mapping between grade, phenotype, and treatment strategy rather than capturing spurious correlations.

Building upon reliable associations at the single-feature level, the coupling effects between imaging features and clinical biomarkers within the CNN–Transformer multimodal representation space are further analyzed, as shown in Table 5. The results demonstrate that when texture heterogeneity captured by 3D ResNet-18 co-occurs with EGFR mutation in GIII tumors, a targeted-therapy-sensitive subtype is consistently identified, with a high objective response rate in the patient cohort. Conversely, the combination of extensive necrosis and high PD-L1 expression is associated with an immune-resistant subtype, reflecting the interaction between hypoxic microenvironments and immune evasion mechanisms. For GII tumors, the coupling of enhancement uniformity and AFP level corresponds to favorable chemotherapy response. These findings indicate that grade-guided collaborative modeling strengthens high-order cross-modal interactions through conditional constraints, enabling the formation of clinically meaningful clusters in the joint feature space. Notably, the high concordance between non-invasive grading predictions and pathological grading further confirms that the identified mechanisms are grounded in stable structural representations rather than noise. Overall, these experiments validate the effectiveness of the proposed approach at the mechanistic level by demonstrating that grading-guided multimodal modeling yields interpretable, verifiable, and clinically actionable treatment response patterns, thereby providing reliable support for precision treatment decision-making.

### 4.6. Ablation Study

To systematically verify the effectiveness of the proposed framework and quantify the independent contribution of each core component, we conducted a comprehensive ablation study. By progressively integrating multimodal inputs, deep feature fusion architecture, and the grade-guided collaborative mechanism, we evaluated the performance gains at each stage relative to the baseline models. This stepwise validation aims to demonstrate that the performance improvements are not merely due to increased model complexity, but rather stem from the specific structural designs that capture the intrinsic associations between tumor grading and treatment response.

As presented in Table 6, the experimental results illustrate a consistent upward trend in performance with the addition of each module. The baseline model, relying solely on imaging features, exhibited limited discriminative capability with an accuracy of 71.4% and an AUC of 0.72. The introduction of clinical phenotypic data in the Early Fusion variant provided a tangible improvement, increasing grading accuracy to 76.8% and verifying the complementary value of multimodal information. Subsequently, replacing the shallow concatenation with the CNN–Transformer architecture for Deep Fusion further enhanced the model’s ability to capture complex cross-modal interactions, raising the AUC to 0.79. However, the most significant performance leap was achieved by integrating the proposed Grade-Guided Collaborative Mechanism. This final configuration maximized the model’s potential, achieving the highest grading accuracy of 84.6% and a Kappa coefficient of 0.81. In terms of treatment response, the proposed framework attained an AUC of 0.85 and a C-index of 0.78, significantly outperforming the unguided Deep Fusion model. These findings confirm that incorporating tumor grade as a structural prior effectively filters irrelevant noise and emphasizes discriminative features, thereby establishing a robust and interpretable link between tumor malignancy and treatment sensitivity.

### 4.7. Validation of the Grade-Guided Structural Constraint

To rigorously verify that the performance gains stem from the valid semantic guidance of tumor grading rather than merely increased model complexity, and to confirm the prognostic value of the identified subtypes, we conducted two targeted validation experiments: randomized label ablation and intra-grade stratification analysis.

#### 4.7.1. Impact of Grading Semantics: A Permutation Test

We designed a “Label Shuffling” experiment to test the hypothesis that the grade-guided fusion mechanism relies on correct biological semantics. In this setup, the pathological grade labels $y^{g}$ fed into the fusion module were randomly permuted across the training batch, destroying the specific correspondence between imaging features and grade while preserving the distribution and dimensionality of the input vectors.

As shown in Table 7, when the grading labels were randomized, the model’s performance significantly deteriorated. The AUC for response prediction dropped from 0.85 to 0.77, and subtype consistency decreased to 0.64, falling even slightly below the “No Guidance” baseline. This result indicates that providing mismatched grading information introduces structural noise that disrupts feature learning. It powerfully confirms that the effectiveness of our framework derives from the correct semantic association between tumor grade and imaging phenotypes, rather than simply the addition of learnable parameters.

#### 4.7.2. Intra-Grade Prognostic Stratification

To address the clinical reality of “different responses within the same grade,” we performed an intra-grade analysis to verify whether our model could identify distinct prognostic subgroups within a fixed grading category. We focused on the High-Grade (GIII) cohort (N=290), which typically presents the greatest clinical management challenge.

The collaborative model stratified the GIII patients into two distinct subtypes: a “High-Risk” group (correlated with texture heterogeneity and necrosis) and a “Low-Risk” group (correlated with high immune infiltration). As presented in Table 8, quantitative survival analysis revealed a statistically significant difference between these two subgroups. The Low-Risk group achieved a median Progression-Free Survival (PFS) of 14.2 months, significantly longer than the 5.8 months observed in the High-Risk group (Log-rank p<0.005). This finding demonstrates that the discovered subtypes possess independent prognostic value beyond standard pathological grading, successfully resolving the heterogeneity that traditional grading alone cannot capture.

### 4.8. Discussion

#### 4.8.1. Mechanism of Feature Interaction and Cross-Tumor Generalization

To further elucidate the internal logic of collaborative modeling, we analyzed how intermediate grading features explicitly regulate the clustering of treatment response subtypes. Structurally, the shared 3D convolutional backbone serves as the fundamental feature extractor, sharing weights across the first four encoding stages to generate a unified high-dimensional spatial tensor *F*. The regulation mechanism is realized through the specific projector and the gated assembly module. Specifically, the grading supervision forces the shared backbone to encode malignancy-sensitive patterns (e.g., necrosis or heterogeneity). These patterns act as a structural prior that drives the generation of the position-wise sampling gate $g_{i}$. This gate dynamically filters the reconstruction residual $\Delta_{i}$, allowing only those local feature perturbations that are structurally consistent with the predicted tumor grade to contribute to the latent clustering vector *u*, thereby preventing noise or irrelevant background variations from distorting the response subtype boundaries.

Furthermore, regarding performance consistency across different pathologies, our stratified analysis reveals distinct behaviors between task specificity and tumor specificity. For the grading task, the model exhibited high consistency across both Non-Small Cell Lung Cancer (NSCLC) and Hepatocellular Carcinoma (HCC) cohorts (Kappa > 0.80), suggesting that the morphological signatures of malignancy—such as infiltrative margins and textural complexity—are shared visual concepts independent of the organ. Conversely, the treatment response prediction demonstrated a degree of tumor specificity: HCC response modeling relied heavily on multi-phase enhancement patterns reflecting vascularity, whereas NSCLC response was predominantly driven by intratumoral texture heterogeneity. Despite these phenotype-level differences, the grade-guided framework successfully aligned these distinct features into stable risk subtypes, confirming that the underlying coupling mechanism between “malignancy degree” and “therapeutic sensitivity” is a generalized biological principle that transcends specific tumor types.

#### 4.8.2. Clinical Significance

This study is grounded in real-world oncological diagnostic and treatment workflows and addresses the long-standing clinical challenge in which tumor grading and treatment response are considered in a fragmented manner. A grading-centered collaborative modeling framework is constructed and validated, with its core value lying in transforming grading information from a traditional static diagnostic indicator into conditional information that continuously influences model behavior within specific architectures. In clinical practice, pathological grading is still regarded as the gold standard; however, its acquisition relies on invasive biopsy procedures, and the results are often constrained by sampling location and intratumoral spatial heterogeneity. Consequently, it is difficult to reflect the global tumor status or to support repeated assessment during treatment. In contrast, non-invasive grading prediction is enabled by leveraging the spatial modeling capability of 3D ResNet-18 for multimodal imaging together with the structured encoding of clinical phenotypes by MLPs, allowing grading information to be continuously obtained before treatment, during therapy, and throughout follow-up. This provides more temporally consistent quantitative evidence for dynamic risk assessment and treatment adjustment.

More importantly, the proposed grade-guided CNN–Transformer collaborative model does not remain at the level of grading prediction alone. Instead, treatment-sensitive and treatment-resistant subtypes are further revealed under grading-conditioned modeling, such that grading is explicitly embedded into the treatment decision logic. In low-grade tumors, subtypes that are highly sensitive to chemotherapy are identified based on relatively homogeneous imaging phenotypes and stable clinical features, thereby supporting standardized treatment strategies and avoiding unnecessary intensified interventions. In high-grade tumors, interactions between imaging heterogeneity and molecular biomarkers are explicitly captured through the CNN–Transformer fusion structure, enabling the identification of patient groups that are more likely to benefit from targeted therapy or immunotherapy. Through this progressive modeling paradigm from grading to subtype and then to treatment mechanism, grading is no longer an isolated classification outcome but becomes a key mediator linking imaging phenotypes, biological mechanisms, and therapeutic strategies, thereby providing more actionable quantitative support for multidisciplinary clinical decision-making.

#### 4.8.3. Generalization Potential

In parallel with clinical value assessment, the generalization ability of the proposed framework across different tumor types is also investigated. Experimental results on independent tumor datasets demonstrate that the framework, built upon multimodal representation learning with 3D ResNet-18 and CNN–Transformer architectures, does not rely on experience patterns specific to a single cancer type. Instead, more structural associations between grading and treatment response are learned. In solid tumors such as hepatocellular carcinoma, which exhibit distinct etiological backgrounds and biological characteristics, high consistency in non-invasive grading and stable treatment response subtypes are still maintained. This indicates that the coupling relationships among imaging heterogeneity, clinical phenotypes, and treatment sensitivity possess a certain degree of cross-tumor commonality. Such stability across tumor types is particularly important for real-world clinical deployment. Clinicians are confronted with highly heterogeneous patient populations rather than idealized, single-distribution datasets. A framework based on mature model architectures, such as 3D ResNet and Transformer, and exhibiting strong generalization capability is therefore more likely to be gradually integrated into routine clinical workflows and to provide value across diverse disease scenarios. At the same time, the observed differences in grading–subtype structures across tumor types offer new perspectives for future research, facilitating the distinction between imaging–clinical association patterns that are shared across tumors and those that are organ- or tissue-specific, thereby enabling more fine-grained investigations in precision oncology.

#### 4.8.4. Limitations and Future Directions

Despite the progress achieved in method design and experimental validation, several limitations regarding data sources, label granularity, and methodological nature must be acknowledged. First, the current study relies on a retrospective cohort from a single institution. Although internal cross-validation demonstrated robustness, the lack of external validation on diverse populations limits the assessment of the model’s generalizability across different scanner protocols and patient demographics. Second, the ground truth for treatment response is based on RECIST 1.1 criteria. While clinically standard, RECIST is morphological and often lags behind metabolic or molecular responses, potentially constraining the model’s ability to capture early, non-morphological signs of therapeutic efficacy. Third, regarding interpretability, while the feature association module identifies key phenotypes linked to subtypes, these findings remain correlational. Without prospective biological validation, causal pathways connecting imaging features to treatment outcomes cannot be definitively established. Furthermore, it is essential to contextualize our approach within the broader landscape of multimodal oncology. Unlike recent state-of-the-art frameworks such as DyAM or Deep Orthogonal Fusion (DOF), which typically employ parallel fusion or general attention mechanisms to integrate heterogeneous data, our framework uniquely positions tumor grade as a structural condition. This grade-guided conditioning allows for the explicit disentanglement of response patterns that are specific to malignancy levels, a capability often absent in generic multi-task learning architectures. This structural advantage enables the model to resolve the “different responses within the same grade” paradox more effectively than unconstrained fusion methods.

Future directions will focus on three key areas to address these limitations and expand the multimodal scope. First, to overcome the static nature of pre-treatment inputs, we aim to incorporate longitudinal imaging data, modeling dynamic temporal changes during therapy as highlighted in recent deep learning studies [73,74,75]. Second, we plan to significantly expand the multimodal spectrum by integrating high-dimensional multi-omics data, such as transcriptomics and proteomics [76,77]. By leveraging the proposed grade-guided attention mechanism to align macroscopic imaging phenotypes with microscopic molecular signatures, we aim to evolve the current framework into a comprehensive radio-genomic platform, thereby deepening the mechanistic understanding of treatment resistance at the molecular level [78,79,80]. Finally, transitioning from retrospective analysis to prospective clinical trials is necessary to validate the model’s actual utility in guiding real-world treatment decisions [81,82]. Overall, these directions provide a clear roadmap for evolving the proposed grade-guided collaborative framework from a correlational analysis tool into a robust clinical decision support system.

## 5. Conclusions

This study was conducted to address a long-standing yet insufficiently resolved challenge in precision oncology, namely the absence of a unified modeling framework and interpretable mechanism linking tumor grading with treatment response. In conventional clinical practice, tumor grading predominantly relies on invasive pathological examinations and is typically utilized as an isolated, static diagnostic outcome, which limits its sustained contribution to individualized treatment selection and therapeutic efficacy assessment. To overcome this limitation, a grade-guided multimodal collaborative modeling framework was proposed. Built upon mature deep learning architectures, including 3D ResNet-18, MLP, and CNN–Transformer, the proposed framework explicitly incorporates grading information into multimodal feature fusion and treatment response modeling, thereby enabling an integrated solution for non-invasive grading prediction, treatment response subtype discovery, and mechanism interpretation. From a methodological perspective, the principal contribution lies in introducing tumor grading as a weakly supervised structural prior to guide multimodal feature fusion, such that joint representations with explicit semantic constraints are formed during the fusion stage. This design effectively avoids the discriminative structure entanglement commonly induced by naive feature concatenation or unconditional fusion strategies in traditional approaches. Through the grade-guided CNN–Transformer collaborative modeling mechanism, discriminative patterns that are highly correlated with tumor malignancy and treatment sensitivity are emphasized in the high-dimensional feature space, while non-informative variations are suppressed. Within the same modeling framework, non-invasive grading prediction and grade-conditioned treatment response subtype identification are accomplished simultaneously. Furthermore, by analyzing internal model representations and cross-modal feature couplings, potential biological transmission pathways linking imaging phenotypes, clinical biomarkers, and treatment outcomes are revealed, substantially enhancing the clinical interpretability of the model outputs. Extensive experimental results from multiple perspectives demonstrate the effectiveness and advantages of the proposed approach. In the tumor-grading prediction task, the grade-guided collaborative model achieved an accuracy of 84.6% and a kappa coefficient of 0.81 on real-world clinical data, significantly outperforming single-modality models based on 3D ResNet-18, clinical MLP models, and conventional early fusion methods. In the treatment response prediction task, the proposed model attained an AUC of 0.85, a precision of 0.81, and a recall of 0.79, while also exhibiting favorable stability and discriminative power in subtype consistency and survival analysis. These results indicate that grade-guided multimodal collaborative modeling not only yields superior predictive performance but also provides reliable support for stratified treatment decision-making and risk assessment in real clinical scenarios.

## Figures and Tables

**Table 1:** Detailed dataset composition, demographics, and clinical characteristics.

Category	Sub-Category	No. of Patients	No. of Samples/Value
***Demographics***			
Total Population	–	500	100%
Age (Years)	Mean ± SD	–	62.4±11.5
	Range	–	34–87
Sex	Male	312	62.4%
	Female	188	37.6%
***Tumor Characteristics***			
Tumor Type	Non-Small Cell Lung Cancer (NSCLC)	265	53.0%
	Hepatocellular Carcinoma (HCC)	235	47.0%
Pathological Grade	Grade I–II (Low/Intermediate)	210	42.0%
	Grade III (High)	290	58.0%
***Imaging Data Acquisition***			
CT Imaging	Non-contrast + Multi-phase	500	1500
MRI Imaging	T1/T2/DCE Sequences	420	1260
PET Imaging	^18^F-FDG PET/CT	310	310
***Treatment & Response***			
Treatment Regimen	Chemotherapy	185	37.0%
	Targeted Therapy	140	28.0%
	Immunotherapy (e.g., PD-1/PD-L1)	95	19.0%
	Radiotherapy	80	16.0%
Response (RECIST)	Responders (CR/PR)	245	49.0%
	Non-Responders (SD/PD)	255	51.0%
Follow-up	Survival Records	500	Median: 28 Months

CR: Complete Response; PR: Partial Response; SD: Stable Disease; PD: Progressive Disease; –: Not applicable.

**Table 2:** Comparison of tumor-grading performance.

Category	Method	Acc. (%)	Kappa
Single-modality grading	3D ResNet-18 (CT)	71.4	0.62
Single-modality grading	MLP (Clinical features)	68.9	0.58
Traditional multimodal fusion	3D ResNet + MLP (Early fusion)	76.8	0.69
Multimodal w/o grade guidance	CNN–Transformer multimodal model	79.2	0.72
Proposed method	Grade-guided CNN–Transformer collaborative model	84.6	0.81

**Table 3:** Comparison of treatment response prediction, subtype stability, and survival analysis.

Category	Method	AUC	Precision	Recall	Consistency	C-Index
Single-modality response	3D ResNet-18 (PET)	0.72	0.69	0.66	0.55	0.66
Single-modality response	GMM (Clinical biomarkers)	0.70	0.67	0.64	0.57	0.65
Traditional multimodal fusion	3D ResNet + MLP (Early fusion)	0.76	0.73	0.71	0.63	0.71
Multimodal	CNN–Transformer w/o grading guidance	0.79	0.75	0.74	0.66	0.73
Proposed method	Grade-guided CNN–Transformer collaborative model	0.85	0.81	0.79	0.78	0.78

**Table 4:** Key imaging and clinical features associated with tumor grading and treatment preference.

Feature Type	Feature	Dominant Grade	Preferred Treatmen
Imaging	Texture heterogeneity	GIII	Targeted therapy
Imaging	Enhancement uniformity	GII	Chemotherapy
Imaging	Necrosis proportion	GIII	Radiotherapy
Imaging	PET metabolic heterogeneity	GIII	Targeted therapy
Clinical	PD-L1 level	GIII	Immunotherapy
Clinical	EGFR mutation	GIII	Targeted therapy
Clinical	AFP level	GII–III	Chemotherapy

**Table 5:** Grading-dependent response subtypes revealed by cross-modal feature coupling.

Cross-Modal Feature	Grade	Response Subtype	Evidence
Texture heterogeneity + EGFR mutation	GIII	Targeted-sensitive	82% responders
Necrosis proportion + PD-L1 high	GIII	Immune-resistant	p<0.01
Enhancement uniformity + AFP level	GII	Chemo-sensitive	p<0.05
Model grading vs pathology	All	High concordance	Kappa =0.81

**Table 6:** Ablation study quantifying the independent contribution of each module to tumor grading and treatment response prediction. Best results are highlighted in bold.

Method Variant	Module Configuration			Tumor Grading		Response Prediction		
	*MM*	*DF*	*GG*	Acc. (%)	Kappa	AUC	Prec.	C-Index
Baseline (Imaging Only)	✗	✗	✗	71.4	0.62	0.72	0.69	0.66
Early Fusion (ResNet+MLP)	✓	✗	✗	76.8	0.69	0.76	0.73	0.71
Deep Fusion (CNN–Trans)	✓	✓	✗	79.2	0.72	0.79	0.75	0.73
**Proposed Framework**	✓	✓	✓	**84.6**	**0.81**	**0.85**	**0.81**	**0.78**

MM: Multimodal Inputs (Imaging + Clinical); DF: Deep Feature Fusion (CNN–Transformer); GG: Grade-Guided Collaborative Mechanism. ✓: Included; ✗: Not Included.

**Table 7:** Comparison of model performance under true grading guidance versus randomized grading labels (Label Shuffling).

Configuration	Grading Prior	Response AUC	Consistency	C-Index
No Guidance (Deep Fusion)	None	0.79	0.66	0.73
**Proposed (True Labels)**	**Correct**	**0.85**	**0.78**	**0.78**
Proposed (Label Shuffling)	Randomized	0.77	0.64	0.71

**Table 8:** Survival analysis statistics for discovered subtypes within the high-grade (GIII) patient cohort.

Subtype Within GIII	N	Median PFS (Months)	1-Year Survival Rate	Log-Rank *p*-Value
High-Risk Group	132	5.8 (95% CI: 4.2–7.1)	42.5%	<**0.005**
Low-Risk Group	158	14.2 (95% CI: 11.5–16.8)	78.4%	

PFS: Progression-Free Survival; CI: Confidence Interval.

## Data Availability

The data presented in this study are available on request from the corresponding author.
